# Short hydrocarbon stapled ApoC2-mimetic peptides activate lipoprotein lipase and lower plasma triglycerides in mice

**DOI:** 10.3389/fcvm.2023.1223920

**Published:** 2023-07-21

**Authors:** Denis Sviridov, Amaury Dasseux, Mart Reimund, Milton Pryor, Steven K. Drake, Zack Jarin, Anna Wolska, Richard W. Pastor, Alan T. Remaley

**Affiliations:** ^1^ Laboratory of Lipoprotein Metabolism, Translational Vascular Medicine Branch, National Heart, Lung, and Blood Institute, National Institutes of Health, Bethesda, MD, United States; ^2^ National Heart, Lung, and Blood Institute, National Institutes of Health, Bethesda, MD, United States; ^3^ Laboratory of Computational Biology, National Heart, Lung, and Blood Institute, National Institutes of Health, Bethesda, MD, United States

**Keywords:** hypertriglyceridemia, APOC2, peptides, lipoprotein lipase, hydrocarbon staples

## Abstract

**Introduction:**

Defects in lipolysis can lead to hypertriglyceridemia, which can trigger acute pancreatitis and is also associated with cardiovascular disease. Decreasing plasma triglycerides (TGs) by activating lipoprotein lipase (LPL) with ApoC2 mimetic peptides is a new treatment strategy for hypertriglyceridemia. We recently described a dual ApoC2 mimetic/ApoC3 antagonist peptide called D6PV that effectively lowered TG in several mouse models but has limitations in terms of drug development. The aim of this study was to create the next generation of ApoC2 mimetic peptides.

**Methods:**

We employed hydrocarbon staples, as well as select amino acid substitutions, to make short single helical mimetic peptides based on the last helix of ApoC2. Peptides were first tested for their ability to activate LPL and then in hypertriglyceridemia mouse models. All-atom simulations of peptides were performed in a lipid-trilayer model of TG-rich lipoproteins to discern their possible mechanism of action.

**Results:**

We designed a single stapled peptide called SP1 (21 residues), and a double stapled (stitched) peptide called SP2 (21 residues) and its N-terminal acylated analogue, SP2a. The hydrocarbon staples increased the amphipathicity of the peptides and their ability to bind lipids without interfering with LPL activation. Indeed, from all-atom simulations, the conformations of SP1 and SP2a are restrained by the staples and maintains the proper orientation of the LPL activation motif, while still allowing their deeper insertion into the lipid-trilayer model. Intraperitoneal injection of stapled peptides (1–5 umoles/kg) into ApoC2–hypomorphic mice or human ApoC3-transgenic resulted in an 80%–90% reduction in plasma TG within 3 h, similar to the much longer D6PV peptide (41 residues). Other modifications (replacement L-Glu20, L-Glu21 with their D-isomers, N-methylation of Gly19, Met2NorLeu and Ala1alpha-methylAla substitutions, N-terminal octanoylation) were introduced into the SP2a peptide. These changes made SP2a highly resistant to proteolysis against trypsin, pepsin, and Proteinase K, while maintaining similar efficacy in lowering plasma TG in mice.

**Conclusion:**

We describe a new generation of ApoC2 mimetic peptides based on hydron carbon stapling that are at least equally potent to earlier peptides but are much shorter and resistant to proteolysis and could be further developed into a new therapy for hypertriglyceridemia.

## Introduction

Lipoprotein Lipase (LPL) is the main enzyme responsible for hydrolyzing plasma triglycerides (TGs) to fatty acids and glycerol (1). Its activity is highly regulated to shunt fatty acids either to skeletal muscle during fasting or to adipocytes after feeding. The differential tissue regulation of LPL activity is accomplished, in part, with proteins that either inhibit (ANGPTL3, ANGPTL4, ANGPTL8, and ApoC3) or activate (ApoA5 and ApoC2) LPL (2). ApoC3 also decreases hepatic uptake of triglyceride-rich lipoproteins (TRL), which include chylomicrons (CM), very low-density lipoproteins (VLDL) and their remnants (3–5). The dysregulation of lipolysis can lead to a marked increase in plasma TG and trigger acute pancreatitis, a highly morbid and potentially lethal condition (6). More moderate elevations in plasma TG from impaired lipolysis have recently been causally associated with atherosclerotic cardiovascular disease (ASCVD) (1, 7–9). New therapies for lowering plasma TG are now a major focus for reducing the relatively large residual ASCVD risk that persists even after effectively lowering low-density lipoproteins with statins (10).

ApoC2 is a small exchangeable apolipoprotein that is mainly expressed in the liver and small intestine (1, 11). It resides on TRL and high-density lipoproteins. The amino-terminal end of ApoC2 forms a large amphipathic helix that facilitates its binding to lipoproteins, which is necessary for it to promote lipolysis (12). It is followed by a random coil region and then a globular alpha helix (Type-G) that activates LPL by an unknown mechanism that may involve a direct protein-protein interaction (12–14). Patients with genetic ApoC2 deficiency can develop severe hypertriglyceridemia and acute pancreatitis (1, 11). These patients are typically treated with a low-fat diet but there is no specific or adequate therapy for this disorder (15).

We recently developed a bi-helical ApoC2 mimetic peptide called D6PV that contains 41 amino acids (16). ApoC2 mimetic peptides could potentially be used as a treatment for pancreatitis in patients with genetic APOC2 deficiency and for the prevention of cardiovascular disease in patients with moderate elevations in plasma triglycerides (1). A single intravenous injection of D6PV markedly lowered TG in ApoC2 hypomorphic mice within a few hours. It also lowered TG in ApoC3 transgenic mice by causing the displacement of ApoC3 from lipoproteins. Except for one amino acid substitution, the second helix of D6PV was identical to the last helical domain of ApoC2 that activates LPL. The first helix of D6PV was based on the flanking random coil region of ApoC2. Six amino acid substitutions were made in this region, so that it could form an amphipathic helix and bind to lipoproteins. A Pro residue was also used to replace an Ala between the two helices of the peptide to improve its solubility (16).

Despite its potent effect in lowering plasma TG, D6PV has several limitations in terms of drug development. Most therapeutic peptides are shorter than 20–30 amino acids. Although the coupling efficiency for solid-phase peptides synthesis is usually at least 95% or better between each amino acid residue, it is nevertheless often difficult and expensive to make very long peptides because of poor yields of the final product (17). In addition, the relatively large number and the type of amino acid substitutions made to D6PV increases the likelihood that it may eventually become immunogenic if used as a chronic therapy. Finally, D6PV contained no modifications to improve its resistance to proteolysis, a key factor in peptide half-life (18).

This report investigates the use of hydrocarbon staples to overcome some of the limitations of D6PV and for making shorter and potentially more effective ApoC2 mimetic peptides. This method utilizes two α,α-disubstituted non-natural amino acids bearing all-hydrocarbon tethers that are inserted into a peptide sequence and are cross-linked with each other (19). They can be placed in adjacent turns of the helix (*i, i *+ 4) to create a “short staple” or by skipping one turn (*i, i *+ 7) for making a “long staple”. Several other peptides with hydrocarbon staples are already being tested in early stage clinical trials (19). We previously showed for ApoA1 mimetic peptides that replacing two amino acids on the hydrophobic face of an amphipathic helix with a hydrocarbon staple stabilizes helix formation and confers proteolysis resistance (20). We also systematically investigated amino acid substitutions and the acylation of apoC2 mimetic peptides for improving their properties.

## Methods

### Peptide synthesis and purification

Peptides were synthesized using standard Fmoc chemistry by Wuxi AppTech (China) or Pepmic Inc. (China) and purified to greater than 95% purity by reverse-phase HPLC. For hydrocarbon stapled and stitched peptides, the ring-closing olefin metathesis (RCM) reaction was carried out on resin-bound peptides, using Grubbs 1st generation catalyst (0.5 eq) in anhydrous DCE (20 ml) added to the resin and agitated with N_2_ for 12 h at 25°C (21). The RCM reaction was repeated twice. The completion of cyclization by RCM was confirmed by test cleavage and LC/MS monitoring.

### DMPC vesicle clearance assay

Dimyristoyl Phosphatidyl Choline (DMPC) (Avanti 850345C) vesicles were made by sonication (0.5 mg/ml) in phosphate buffer saline (PBS). Peptides (0, 25, 50 and 100 µM) in 20 mM TRIS, pH 8.2 were added to DMPC vesicles (0.5 mg/ml) prepared by sonication in PBS and turbidity was monitored in a Victor^3^ plate reader (Perkin Elmer, USA) by monitoring absorbance at 660 nm for 1 h at 24°C. Samples were shaken at 300 RPM. Absorbance over time was plotted and areas-under-the-curve (AOC) were calculated. PBS was used as a negative control sample and Triton X-100 at a final concentration of 1% (w/v) was used as positive control.

### In vitro LPL activity assay

LPL activity was determined by measuring the generation of non-esterified fatty acids (NEFA). In a 96-well plate, 0–2 µM of an ApoC2 mimetic peptide, 0.83 nM of LPL from bovine milk (Sigma-Aldrich), ApoC2-deficient ethylenediaminetetraacetic acid (EDTA)-plasma (diluted with PBS to a final TG concentration of 0.67 mg/dl), 2 IU/ml heparin, 0.2% fatty acids free bovine serum albumin (BSA) (ICN Biomedicals), and PBS (Life Technologies) were combined in a final reaction volume of 50 μl. The reaction mixture was pre-incubated on ice for 30 min and incubated at 37°C for 1 h. NEFA were measured with a coupled enzyme reaction (Wako Diagnostics) in a Synergy H1 microplate reader (BioTek, USA).

### Peptide proteolysis resistance analysis

Peptides (0.1 mM) were incubated at 37°C in 20 mM ammonium bicarbonate buffer at pH 8.2, with 10 ug/ml trypsin or 16 ug/kg Proteinase K. To test resistance to Pepsin, peptides (0.1 mM) were incubated with Pepsin (3 mg/ml) in 0.02M acetate buffer (pH 2). The amount of intact peptide was monitored over time by Matrix-assisted laser desorption/ionization time-of-flight (MALDI-TOF) on a Bruker Autoflex III instrument (Bruker Optics, Inc., USA). Internal standards (Bombesin, ACTH (1–17), ACTH (18–39) and Insulin) were used to quantify the relative amount of peptide in each sample.

### Isothermal titration calorimetry analysis

Isothermal titration calorimetry (ITC) measurements, which depends upon the initial rate of reaction, were performed to assess peptide activation of LPL in nearly undiluted human plasma as previously described (22, 23). Peptides in 20 mM TRIS, pH 8.2 at the indicated concentrations were added to a normolipidemic EDTA-plasma pool. The reaction was initiated by three sequential injections of 1 nM human LPL. The heat of the reaction was monitored with the iTC200 calorimeter (GE Healthcare, USA) and the initial reaction rates at 25°C were converted into relative activity units.

### Circular dichroism spectroscopy analysis

Circular dichroism (CD) spectra were acquired on a Chirascan Q100 spectrometer. CD spectra were recorded in the190–250 nm range, using a 0.1 cm quartz cell and analyzed with Bettsele® on-line software (24).

### Animal procedures and handling

ApoC2–hypomorphic mice (*Apoc2*^h/h^) (25) and human ApoC3-transgenic (hAPOC3-tg) mice (26) were fed a regular rodent chow diet (NIH-31 chow diet: Zeigler Brothers Inc.). Peptides dissolved in 20 mM TRIS (pH 8.2) or vehicle control were injected intraperitoneally or intravenously at the indicated concentrations. Blood samples were collected from the retro-orbital sinus with a heparinized capillary tube. Whole blood was centrifuged at 1,000 g for 20 min at 4°C to obtain plasma. All animal procedures were approved by the National Heart, Lung, and Blood Institute Animal Care and Use Committee (protocol #H-0050).

### Molecular dynamics simulations

Following our previous modeling of ApoC2 and D6PV, molecular dynamics (MD) simulations of a trilayer of POPC and triolein (TO) were used to model the surface of TRL and to capture the behavior of two of its major components: phospholipids (PLs) and TGs (16). The generation of initial coordinates and the long timescale equilibration of the trilayer structures has been noted previously (27). To overcome these barriers, trilayers were first equilibrated at coarse-grained (CG) resolution, using the Martini v2.2 force field with elastic network restraining the peptide structure (28, 29) and the additional 5th hydrophobic bead the tails of the TO to increase hydrophobicity. A symmetric bilayer system was built using the CHARMM-GUI bilayer builder and composed of 160 POPC, 128 TO, 4,139 water, 150 mM NaCl, and two copies ApoC2 59–79 (30, 31). CG simulations were run using the Gromacs 2020.3 MD Software (32–35). The CG trilayer was equilibrated using the CHARMM-GUI Martini Membrane builder equilibration scheme (31). A 1 µs long production simulation used 20 fs timestep. Nonbonded interactions were cutoff at 1.1 nm using the reaction-field electrostatics and potential-shift van der Waals. This simulation was held at 0 surface-tension using the semiisotropic Parrinello-Rahman thermostat (36, 37) with pressure coupling of 12 ps and velocity rescaling (38) with a temperature coupling of 1.0 ps. The CG trilayer was subsequently backmapped (39) to the all-atom resolution, solvated with 12,000 water molecules and 150 mM KCl, and equilibrated using the CHARMM-GUI bilayer equilibration protocol (40) to produce a stable trilayer with the ApoC2 59–79 (P8) peptide. All-atom simulations used the CHARMM36 force field (41, 42), CGenFF to describe missing parameters (e.g., N-Methyl Glycine) (43, 44), and TIP3P water model (45, 46). The system was duplicated, and peptides were modified as necessary (i.e., acylated, stapled, mutated) using the CHARMM software package (47) to produce initial configurations for the SP1 and SP2a peptide systems.

The three all-atom trilayer systems were simulated for 100 ns locally using OpenMM v7.7 (48) for all-atom equilibration prior to running the production simulations on Anton 2 Supercomputer (49). OpenMM simulations were run using a 2 fs timestep, and maintained at 310 K using the Nose-Hoover thermostat (50–52) and 0 surface tension Monte Carlo Membrane Barostat (53). Nonbonded interactions were cutoff at 1.2 nm using particle mesh Ewald method (54) for electrostatics and a 0.8–1.2 nm force-switch for van der Waals. The endpoint of the 100 ns simulation is the initial state shown in Supplementary Figures S3–S5. The three systems were each run for five microseconds on the Anton 2 Supercomputer using a 2 fs timestep in a Multigrator framework (55) at 310 K and 0 surface tension.

All-atom trajectories were analyzed using in-house python scripts using Numpy (56), MDTraj (57), and Freud (58). Timeseries of hydrogen bonds were calculated using wernet_nillson function in MDTraj (59). Residue-wise coordination number were calculated by finding the maximum $s=\sum [1-{\left(r_{i}/r_{0}\;\right)}^{6}]/[1-{\left(r_{i}/r_{0}\right)}^{12}]\;$ where $r_{i}$ is distance between all peptide atoms and the atoms of a given triolein molecular and $r_{0}$ is 0.4 nm. Plots were made using Matplotlib (60). Snapshots were made using VMD software (61).

### Antigenic peptides prediction

Antigenic peptides prediction was performed using the online tool from Universidad Complutense Madrid http://imed.med.ucm.es/Tools/antigenic.pl. The algorithm used in this tool is derived from the method of Kolaskar and Tongaonkar (62) where predictions reflect occurrence of amino acid residues in experimentally known segmental epitopes to predict peptide segments that are likely to be antigenic. The average propensity for each overlapping 7-mer was calculated and a score was assigned to the middle residue (i + 3). Next, the 8-mer peptides with an assigned score more than 1 in each residue were predicted to be antigenic.

### Statistical analysis

Two-way ANOVA test and Tukey's post-test were used to compare means at different time points or peptide concentrations vs. vehicle control group for the indicated experimental studies. Analysis was performed with GraphPad PRISM version 9.5.1 software.

## Results

### D6PV peptide Ala-scanning array

**Figure 1:**
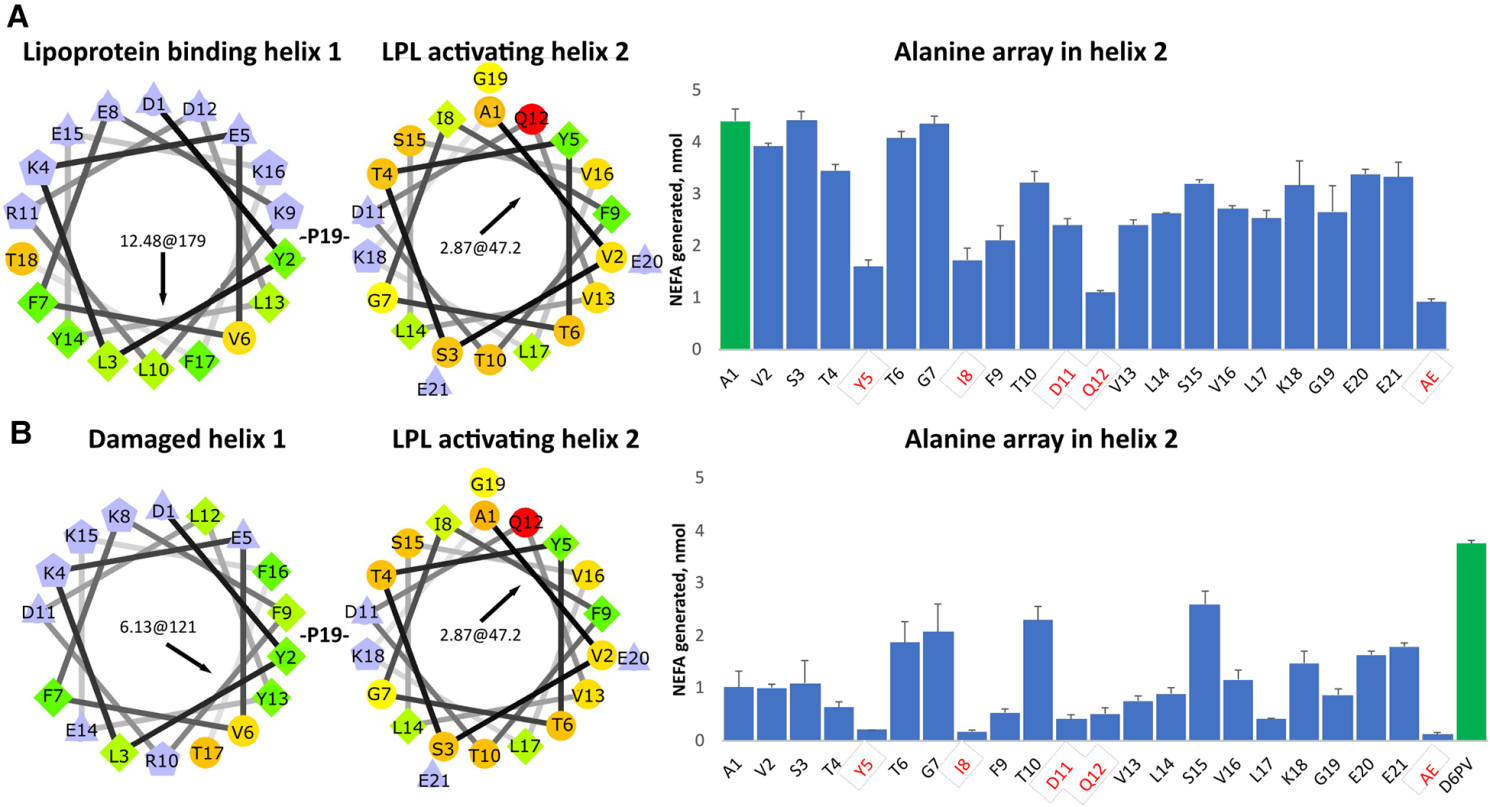
Ala- scanning array of D6PV peptide. (**A**) Helical wheel plots of the two helices of D6PV are shown. Helical wheel color codes: lilac—charged amino acids; orange or red circle—polar; green rhombus or yellow circle—hydrophobic; light yellow—Gly. First numbers in the center of the helical wheel indicate hydrophobic moment, and second number and black arrow indicate angle of hydrophobic moment. On the right graph, each amino acid in the sequence of the second helix were individually replaced with Ala and peptides were tested for LPL activation at a final concentration of 1 uM, using apoC-2 deficient plasma as a substrate (TG final concentration of 3.4 mg/dl). Residues highlighted in red were previously reported as essential for LPL activation. For the AE peptide all four of these amino acids were simultaneously replaced with Ala. First peptide (green bar) has same sequence as D6PV. (**B**) First helix of D6PV was altered (E8 deletion) to decrease its amphipathicity resulting in lower lipoprotein affinity. Ala scan was performed in second helix and peptides tested for LPL activation as described in panel A. Results are shown as mean ± 1SD of triplicates.

To determine the importance of the amino acid residues in the second helix of D6PV in the ability of the peptide to activate LPL, we created a peptide array in which each residue in the second helix was replaced with the amino acid Ala (Figure 1A). Ala is only slightly hydrophobic and tends to promote helix formation regardless of its location in a helix, because its small side chain does not interfere with hydrogen bonding across the peptide backbone (63). As previously described from site directed mutagenesis of the ApoC2 protein (64), Y5, I8, D11 and Q12 were all important in the activation of LPL by the D6PV peptide. The simultaneous replacement of these 4 residues with Ala resulted in a peptide (AE) with the lowest ability to activate LPL. We also observed, the replacement of several strongly hydrophobic amino acids in the second helix (F9, V13, L14, V16, L17) had a greater damaging effect in LPL activation than replacement of the more polar residues (T6, G7, T10, S15, K18, E20, E21), which in most cases did not substantially alter LPL activation. This would be expected because replacing the most hydrophobic groups with Ala would reduce the hydrophobic moment of the helix.

In a second Ala-array (Figure 1B), a single amino acid substitution was also made in the first helix. The amino acid E8 was deleted, which results in a change in the position of the other amino acids with respect to each other on the helical wheel plot (Figure 1B). The modified or “damaged” first helix has approximately half the hydrophobic moment as the original helix and would be expected to have decreased ability to bind to lipids. In general, all the peptides with the damaged first helix in the second array were less active in activating LPL and again the same 4 residues (Y5, I8, D11 and Q12) were the most important. We also observed again that the replacement of hydrophobic amino acids in the second helix (F9, V13, L14, L17) with Ala reduced LPL activation but to a greater degree compared to the first array. Interestingly, substituting the relatively more polar or less hydrophobic amino acids (T6, T10, G7) located near the hydrophobic face of helix with Ala enhanced activation when compared to first control A1 peptide in the array. Replacement of S15 with Ala, which promotes overall helix formation, near the polar face of the peptide also increased LPL activation. Together these findings suggest that when the first helix has reduced lipid affinity, the second helix can also promote lipoprotein binding and help restore LPL activation, if it contains amino acid substitutions that either increase its hydrophobic moment and or helicity.

### Stabilization of peptides by hydrocarbon stapling

Given the necessity of apoC2 peptides to bind lipoproteins for LPL activation, we investigated the use of hydrocarbon staples to design shorter peptides that can still interact with lipoproteins and activate LPL. Based on an antigenicity prediction software, part of the first helix of D6PV (KEVFEKLRDLY) was also predicted to be potentially immunogenic unlike the native sequence (Supplementary Figure S1), thus we also wanted to investigate whether we could make a shorter peptide not containing this sequence.

**Figure 2:**
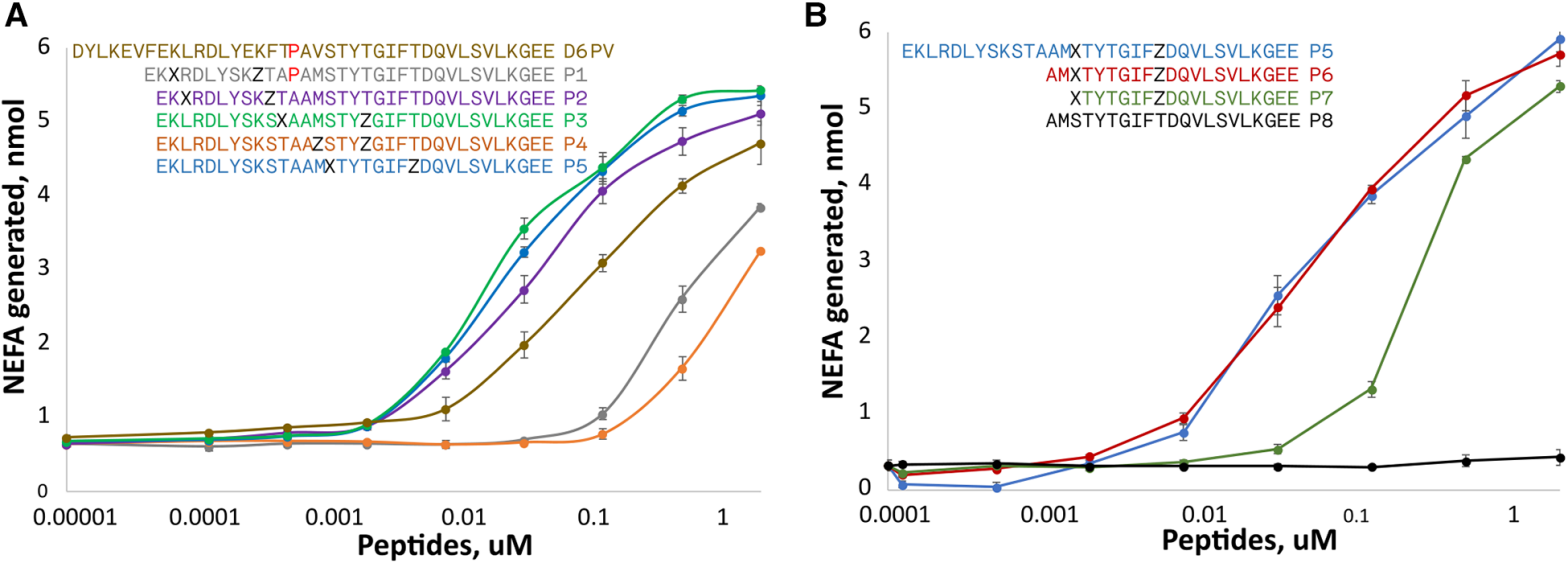
Peptide dose response curves for activation of LPL. Peptides were tested at different doses for *in vitro* LPL activation as described in Figure 1. The color code of the peptide sequences (see legends) are matched to the color of the dose response curves. Amino acid identity are indicated by single letter abbreviation. X indicates position of (**R**)-α-methyl, α-octenyl-glycine and Z indicates position of (**S**)-α-methyl,α-pentenyl-glycine and are the two amino acid substitutions made to introduce the two different arms of the hydrocarbon staples. (**A**) Hydrocarbon staples placed in either first or second helix of apoC2 mimetic peptides containing 33 residues. (**B**) Hydrocarbon staples placed in the second helix of apoC2 mimetic peptides containing 19–21 residues. Results are shown as mean plus ± 1SD of triplicates.

Two amino acids substitutions in the hydrophobic face of either the first or second helix were made to create either a short (spanning 1-turn) or long (spanning 2-turns) hydrocarbon staple in peptides shorter than D6PV from deletion of the first 7 N-terminal amino acids (Figure 2A). A peptide (P1) with an extra Pro between the two helices and a long hydrocarbon staple placed between position 3 and 10 had reduced ability to active LPL compared to D6PV. A second peptide, P2, with the same hydrocarbon staple as P1 but without the extra Pro between the two helices was more active than D6PV. Similarly, the P3 peptide, with a long staple that spanned the two helices (position 11 and 18) was also more potent than D6PV. In the P4 peptide, a short staple between position 14 and 18 was added in the second helix, but it had reduced activity. For the P5 peptide, a long staple in the second helix (position 15 and 22) was added to span the first Gly residue to help stabilize helix formation. It showed relatively good activity and was comparable to P3 in LPL activation.

Next, we investigated the use of hydrocarbon staples in even shorter peptides (21 amino acids) that were just based on the C-terminal helical domain of ApoC2 (second helix) (Figure 2B). The P6 peptide that had a long hydrocarbon staple in the same position as P5 and was just as active, although it was 12 amino acids shorter than P5. In P7, two additional amino acids were removed from the N-terminus but this reduced its potency. The P8 peptide, which is just based on the native sequence of ApoC2 (residues 59–79) and does not contain any hydrocarbon staples, was completely inactive.

For the most active peptides, we observed saturation in the rate of lipolysis with increasing peptide concentration. The V_max_ of the reaction, however, did not appear to substantially differ between the most effective peptides, but we did observe differences in their EC_50s_. The most potent peptides in stimulating lipolysis (P3, P5, P6) had EC_50s_ of 0.025 to 0.033 uM, whereas D6PV had an EC_50_ of 0.107 uM.

### Peptide design strategy for conferring resistance to proteolysis

**Figure 3:**
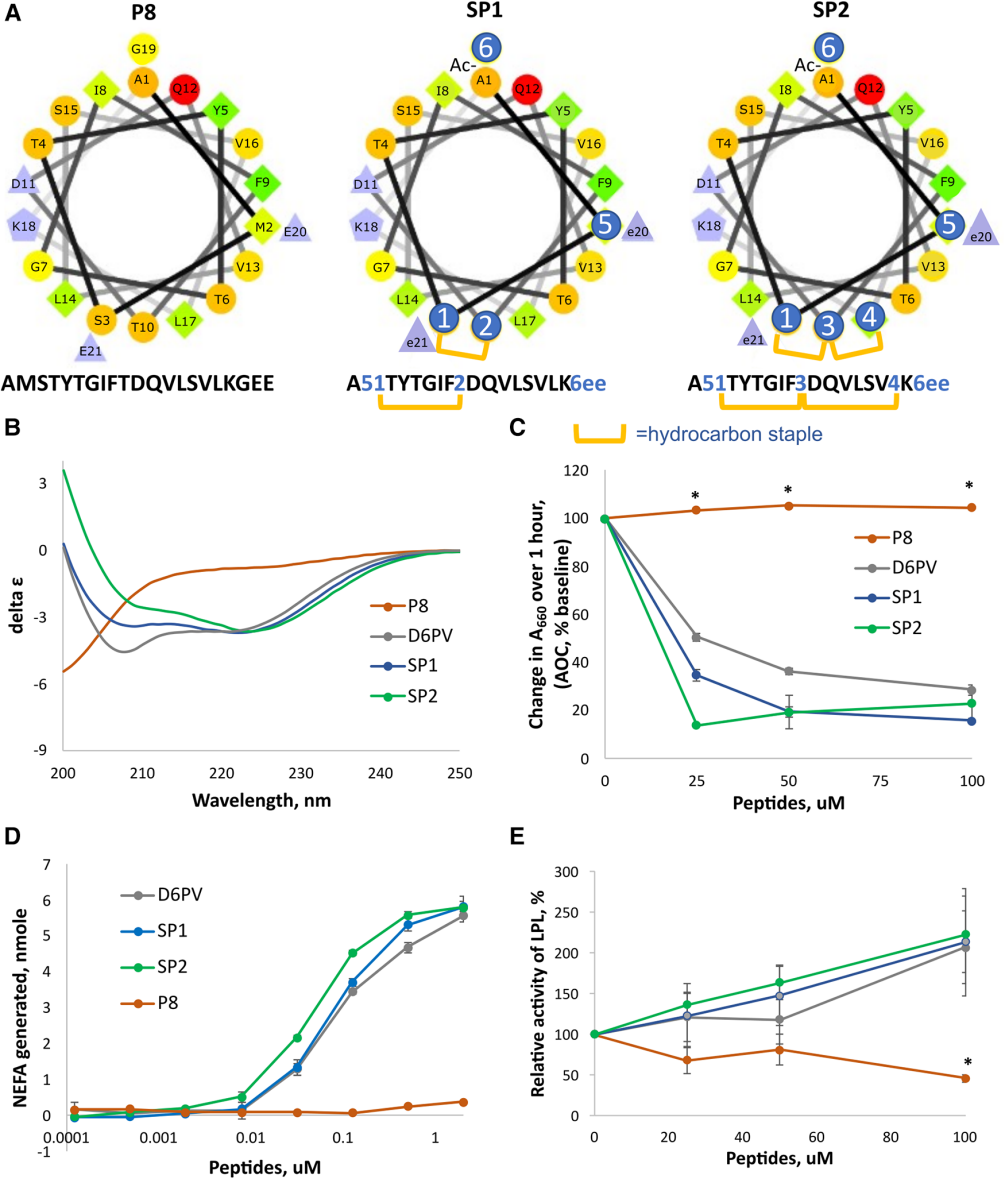
Design and properties of stapled peptides. (**A**) Helical wheel plots of native ApoC2 (59-–79) fragment (peptide P8) and SP1 and SP2 peptides. Changes to native sequence marked with blue circles: 1 = (R)-α-methyl,α-octenyl-glycine (R8); 2 = (S)-α-methyl,α-pentenyl-glycine (S5); 3 = bis-pentenyl-glycine (B5); 4 = (S)-α-methyl,α-octenyl-glycine (S8); 5 = Norleucine; 6 = N-methyl-glycine; e = D-glutamate; hydrocarbon staple is between R8 and S5 or between R8-B5-S8 residues for stiched peptides as shown in yellow bars. (**B**) CD spectrum of peptides. Double minima at 208 nm and 222 nm in D6PV, SP1 and SP2 peptides spectra indicate alpha-helical secondary structure calculated to be 42, 40 and 46% respectively. P8 has a spectral characteristic of a random coil. (**C**) DMPC vesicle solubilization of peptides. (**D**) LPL activation assay of peptides as done in Figure 2. (**E**) LPL activity assay with pooled human plasma measured by isothermal titration calorimetry as described in methods. Results represent the mean ± 1SD of triplicates. *p < 0.05 (P8 vs. D6PV, SP1 and SP2).

Besides stabilizing helix formation, hydrocarbon staples also protect peptide bonds within the stapled region from cleavage by endoproteases (65), which can be a major determinant of peptide half-life (66, 67). To also protect against inactivation by aminopeptidases and carboxypeptidases, we also modified the amino and carboxy terminal regions of the P6 single stapled peptide shown in Figure 2 to produce the SP1 peptide (Figure 3). The N-terminus was acetylated, which blocks aminopeptidases (68). The last two L-glutamic acid residues at the C-terminus were changed to D-glutamic, which blocks degradation by carboxypeptidases, because most proteases are stereospecific (69, 70). To protect K18 from trypsin cleavage, we tried to replace it with its D-isomer but this significantly decreased its activity (Supplementary Figure S2). Instead, we replaced the preceding amino acid in the sequence, G19, to N-methyl-Gly (sarcosine). Trypsin cuts after Lys residues and N-methylation of the peptide bond at this position confers trypsin resistance (70). Finally, we replaced Met in position 2 with the isosteric non-natural amino acid NorLeucine to make the peptide resistant to oxidation. As shown in Supplementary Figure S2, the new SP1 peptide with all these amino acid substitutions was similar to P6 in its potency to activate LPL.

To further enhance resistance to proteolysis, we also investigated the addition of a second long hydrocarbon staple to the last helix of ApoC2 (Figure 3A). Peptides with more than one hydrocarbon staple are called stitched peptides and are highly helical (19). By CD spectroscopy, the stitched peptide SP2 was slightly more helical than the single staple SP1 peptide or D6PV, whereas the control peptide P8 based on the native ApoC2 sequence was mostly in a random coil configuration (Figure 3B). These peptides were also tested in a functional assay for lipid binding by determining their ability to bind to and solubilize DMPC vesicles (Figure 3C). Consistent with the CD spectroscopy results, SP2 also appeared to be the most effective in solubilizing DMPC vesicles, whereas the control P8 peptide was inactive. By the *in vitro* LPL activation, SP2 appeared to be slightly more potent than SP1 than SP2 or D6PV for LPL activation (Figure 3D). Isothermal titration calorimetry, which kinetically measures the initial reaction rate and uses nearly undiluted plasma as a substrate, was also used to assess the peptides, because it better reflects the *in vivo* conditions for lipolysis (22). By this method both the new stapled peptides and D6PV doubled the activity of LPL on top of endogenous activators (Figure 3E). In contrast, the control P8 peptide did not increase LPL activity.

### Susceptibility of peptides to proteolysis

**Figure 4:**
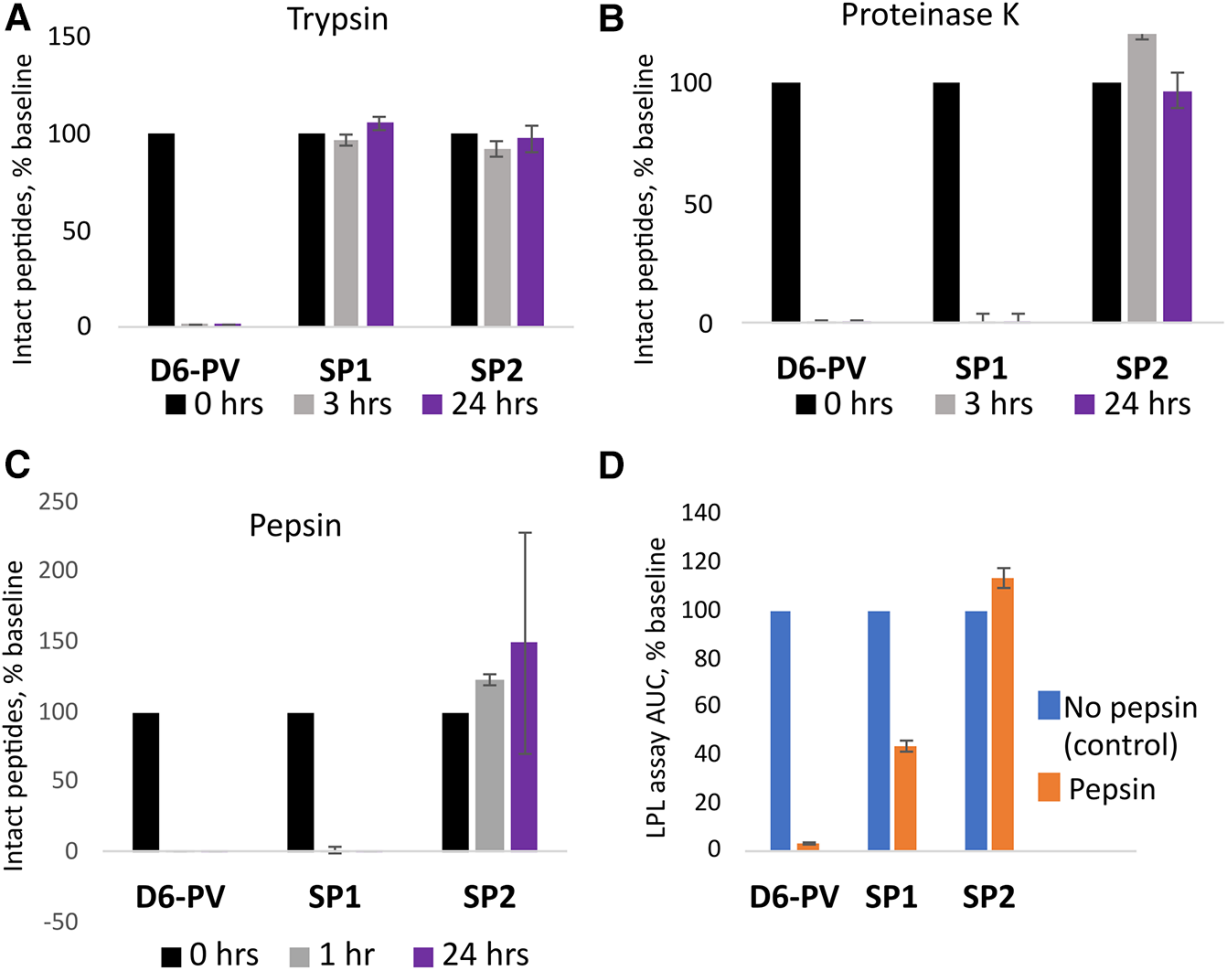
Peptide proteolysis resistance. Peptides were incubated with trypsin (**A**) or proteinase K (**B**) at pH 8.0 or pepsin (**C**) at pH 2.0 as described in methods. Graphs indicate the percent of original amount of full-length peptide detected by MALDI-TOF MS after incubation period. (**D**) After 1 h incubation with pepsin, the reaction mixture was adjusted back to pH 8.0 and the *in vitro* LPL activation assay was performed (**D**). The results are compared to the activity of the peptides without pepsin treatment as a percent of the area-under-the-curve. Results represent the mean ± 1SD of triplicates.

Peptide resistance to proteolysis was tested by incubating peptides with trypsin, and proteinase K, followed by MALDI-TOF analysis for detecting full-length peptides after various timepoints (Figure 4). As expected, D6PV was quickly degraded by both protease and essentially no intact peptide was detected even after the first 3-hour time point. In contrast, SP1 was almost completely resistant to trypsin but not Proteinase K (Figure 4A), which has broad specificity and cleaves after aliphatic and aromatic amino acids (71). SP2 was resistant to both proteases, presumably because of the longer peptide sequence covered by the two hydrocarbon staples (Figures 4A,B).

The peptides were also incubated with pepsin, a broad specificity gastric protease with an acidic pH optimum (72). After incubation with pepsin at pH 2, only SP2 was resistant to proteolysis. No intact peptide was observed for either D6PV or SP1 after 1 h of pepsin treatment. We also tested the samples after pepsin treatment for their ability to activate LPL after adjusting the pH back to 7.4 to inhibit pepsin. Consistent with the MALDI-TOF analysis, SP2 was fully active in activating LPL after the pepsin treatment, whereas D6PV was almost completely inactive. SP1 still showed some residual ability to activate LPL after the pepsin treatment and pH neutralization, possibly due to the ability of SP1 fragments to partially activate LPL.

### *In vivo* effect of peptides on hypertriglyceridemia

**Figure 5:**
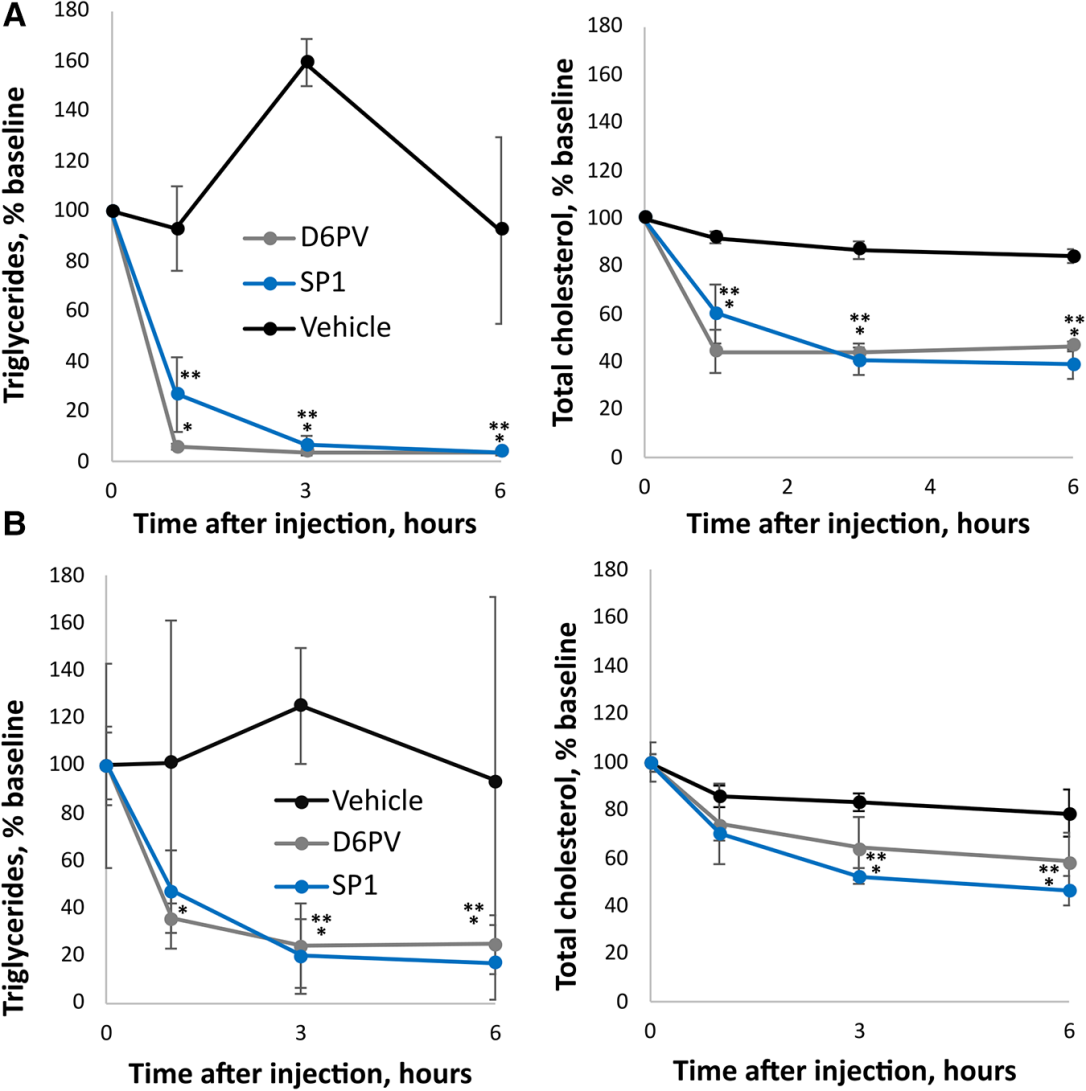
Effect of SP1 peptide on lipids in mice. Mice underwent a single dose IP injections of SP1, D6PV or were treated IP with vehicle control and at the indicated time intervals plasma was collected and analyzed for triglycerides (TG) and total cholesterol (TC). Data are presented as percent baseline. (**A**) *Apoc2*^h/h^ mice (*n* = 4, 3.8-months old) were injected with 1 umole/kg of peptides (D6PV 4.7 mg/kg, SP1 2.4 mg/kg). Baseline TG was 1,522 +/− 765 mg/dl, and baseline TC was 125 +/− 27 mg/dl. (**B**) hApoC3-TG mice (*n* = 4, 5.5 months old) were injected with 5 umole/kg of peptides (D6PV 23.4 mg/kg, SP1 12 mg/kg). Baseline TG was 5,544 +/− 2,854 mg/dl, and baseline TC was 152 +/− 26 mg/dl. Results represent the mean ± 1SD. *p < 0.05 (D6PV vs. Vehicle), **p < 0.05 (SP1 vs. Vehicle).

As shown in Figure 5, the SP1 peptide was tested for its effect on plasma triglycerides and cholesterol in two different mouse models of hypertriglyceridemia. In *Apoc2*^h/h^ mice, both SP1 and D6PV showed a similar ability to lower plasma triglycerides when used at the same molar dose. There was more than a 90% reduction in plasma triglycerides for both peptides 3 h after IP injection. **Notably,** both peptides also lowered plasma cholesterol but were less effective compared to triglyceride reduction likely because TG-rich lipoproteins contain less cholesterol than triglycerides (16). We also tested the peptides in hAPOC3-Tg mice (Figure 5B). Again, both peptides were similar in their ability to lower plasma triglycerides and cholesterol, although the percent reductions for both lipids were not as great as seen in the *Apoc2*^h/h^ mice.

**Figure 6:**
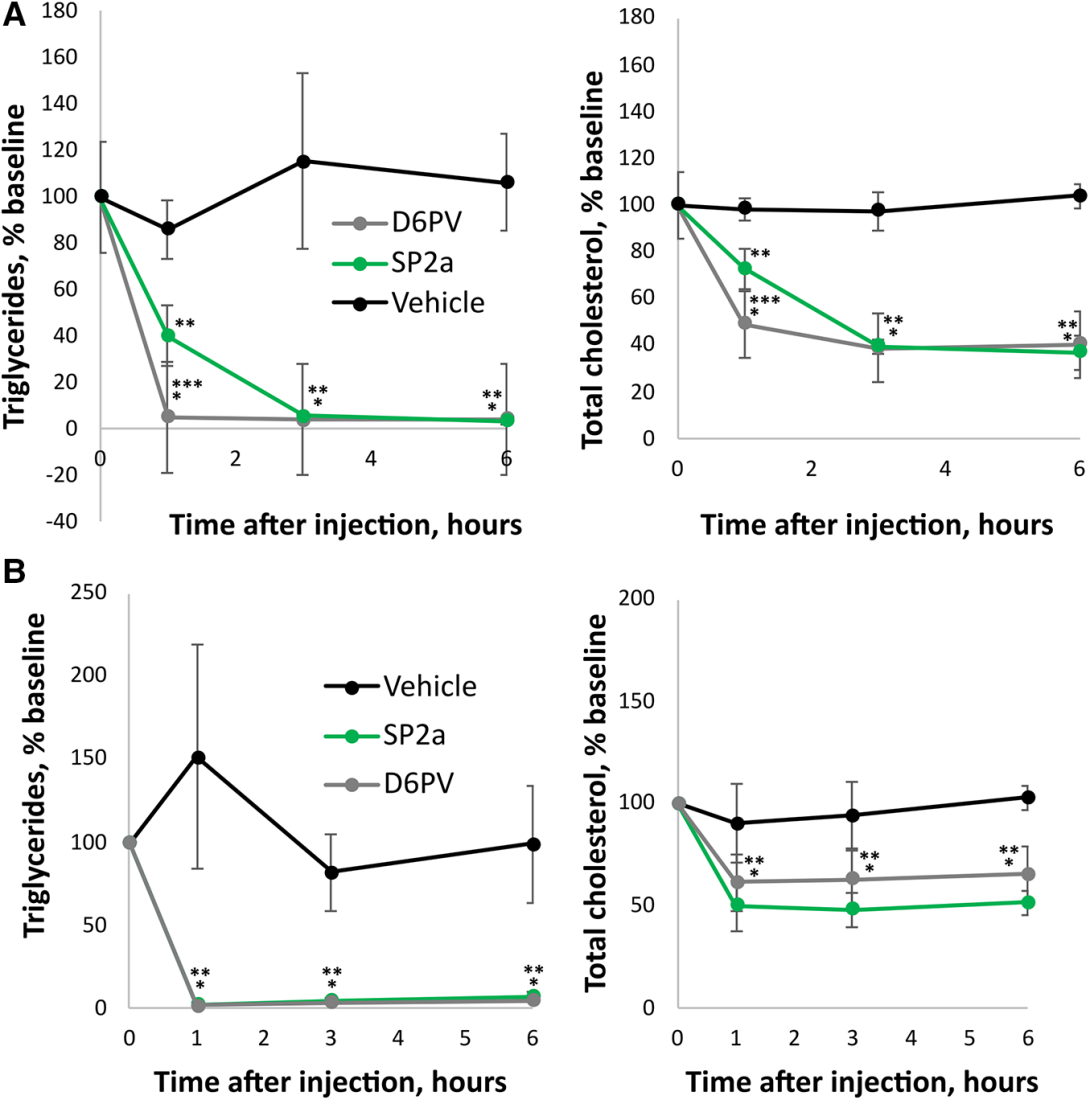
Effect of SP2a peptide on lipids in mice. Mice underwent a single dose (1 umole/kg; D6PV 4.7 mg/kg, SP2a 2.6 mg/kg) IP injections of SP2a, D6PV or vehicle control and at the indicated time intervals plasma was collected and analyzed for triglycerides (TG) and total cholesterol (TC). Data are presented as percent baseline. (**A**) *Apoc2*^h/h^ mice (*n* = 5, 7–8 months old). Baseline TG was 2,713 +/− 2,072 mg/dl, and baseline TC was 141 +/− 53 mg/dl. (**B**) *Apoc2*^h/h^ mice (*n* = 4, 11 months old) underwent a single dose (1 umole/kg; D6PV 4.7 mg/kg, SP2a 2.6 mg/kg) IV injections of SP2a, D6PV or vehicle control. Baseline TG was 2,033 +/− 577 mg/dl, and baseline TC was 92 +/− 16 mg/dl. Results represent the mean ± SD, *p < 0.05 (D6PV vs. Vehicle), **p < 0.05 (SP2a vs. Vehicle).

Next, we compared D6PV to a stitched peptide called SP2a for its ability to lower plasma triglycerides and cholesterol in *Apoc2*^h/h^ mice (Figure 6). SP2a is similar to SP2 in design but its N-terminus is modified with an octanoic acid instead of acetic acid. The first Ala residue of SP2 is also alpha-methylated (amino-isobutyric acid). These changes markedly increased the yield of SP2a compared to SP2 during solid phase synthesis, allowing us to obtain sufficient amounts of the peptide to perform animal studies. Like SP2, SP2a was highly helical by CD spectroscopy, resistant to proteolysis and was also more potent than SP1 and D6PV in the LPL activation assay (Supplementary Figure S3). When injected IP into mice, SP2a lowered plasma triglycerides like D6PV at the 3-hour time point but appeared to be possibly less effective at the 1-hour time point. The addition of octanoic acid to the N-terminus likely leads to the oligomerization of the peptide (73), which may have delayed the transport of the larger oligopeptide complex into the plasma compartment after IP injection into the peritoneal cavity (74). We, therefore, investigated the direct intravenous injection of SP2a and D6PV (Figure 6B). By this route of administration, both peptides appeared to lower plasma TG faster and they showed similar effects at all time points.

### Molecular dynamics simulation of peptides

**Figure 7:**
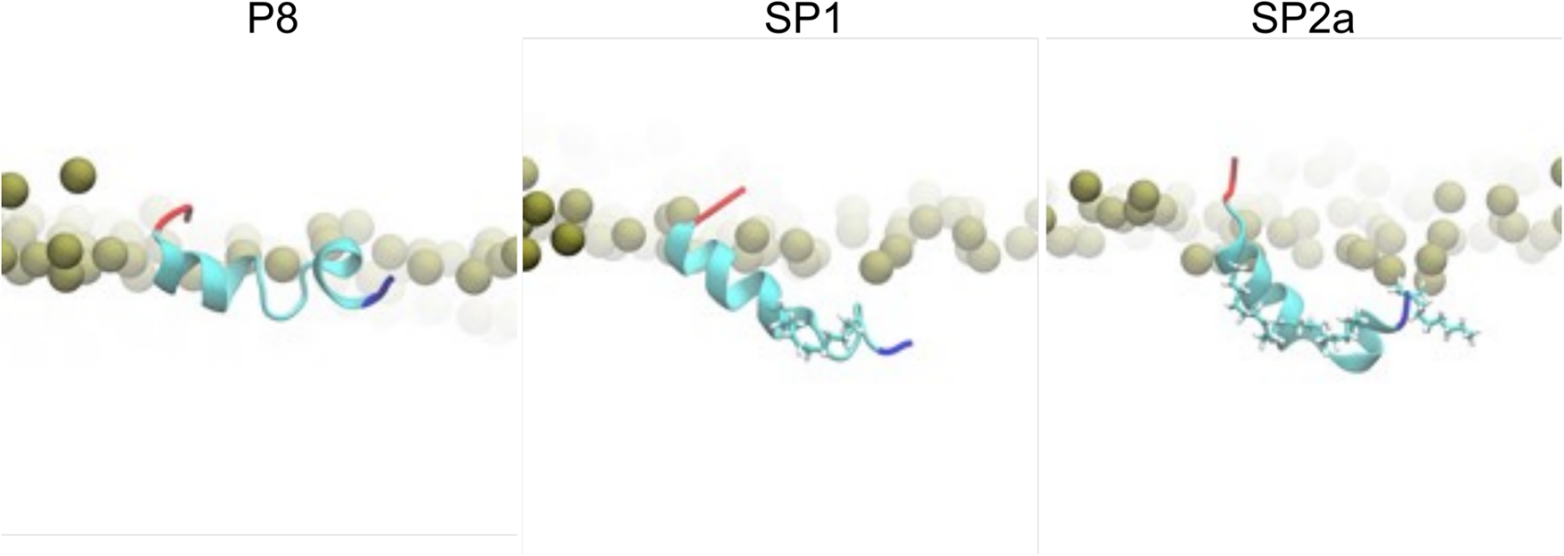
Molecular dynamic simulation of peptides. Zoomed-in snapshots of P8, SP1, and SP2a interacting with a trilayer of PL and TG after 5 microseconds. Coloring: Peptide N- and C-termini blue and red, respectively, with remaining residues cyan; carbons and hydrogens of peptide staple and acylation cyan and white, respectively; nearby phosphorus atoms of PL gold spheres.

To gain further insight into the potential mechanism of action of the peptides, all-atom molecular dynamic simulations of native ApoC2 59–79 (P8), stapled SP1, and stitched SP2a mimetic peptides were simulated on the surface of a model TRL. Initially, all three simulation systems for the peptides were started with the peptides in a helical state and the acidic C-terminus was the least buried region of the peptides. Quickly into the simulations, however, a helical break occurs near the first Gly residue for all the peptides. As can be seen in Figure 7, the native P8 peptide (ApoC2 residues 59–79) inserts to about the phosphate plane in the phospholipid monolayer. Even though the modified stapled peptides form a similar break in their helical structure as P8, these peptides form a wedge shape with the vertex at the helical break and they insert more deeply into the phospholipid monolayer. Not unexpectedly given their hydrophobic constituents, the hydrocarbon staple of the modified peptides faced toward the TG core of the trilayer throughout the simulation.

**Figure 8:**
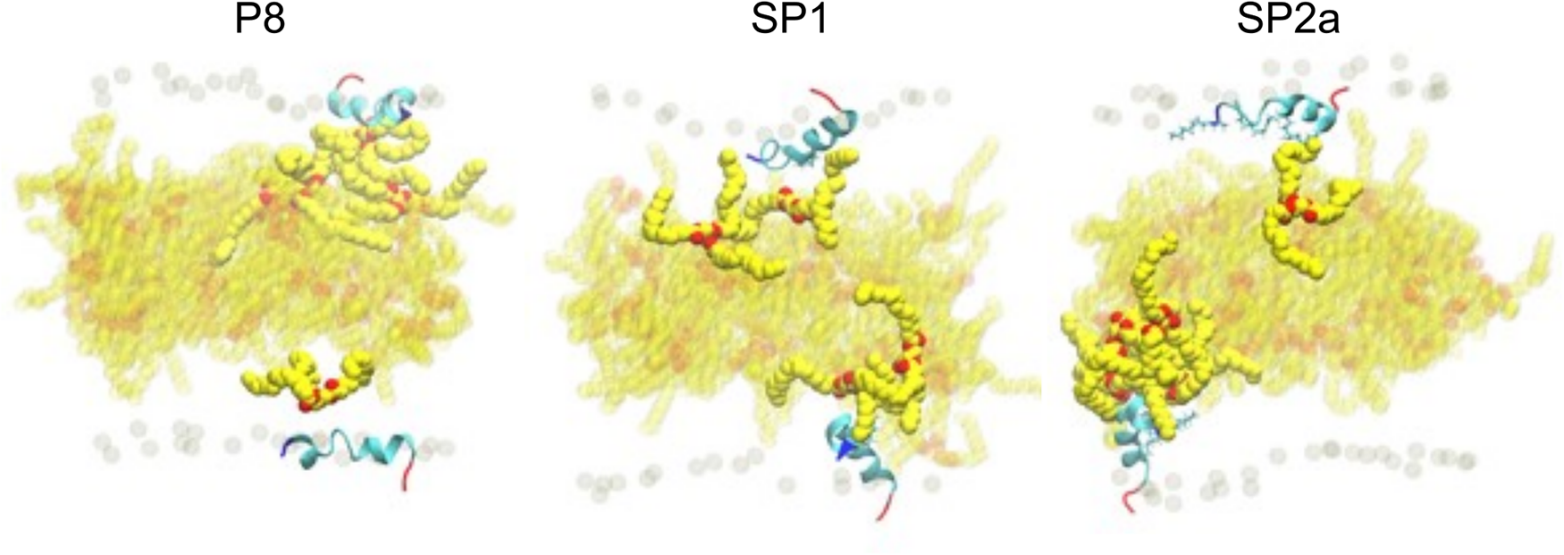
Molecular dynamic simulation of peptide interaction with TG. Snapshots of P8, SP1, and SP2a interacting with a trilayer of PL and TG after 5 microseconds. Peptide and phosphorous atom coloring as for Figure 7. Carbon and oxygen of TG in yellow and red spheres, respectively. TG with any atom near peptide is opaque and background TG are semi-transparent.

**Figure 9:**
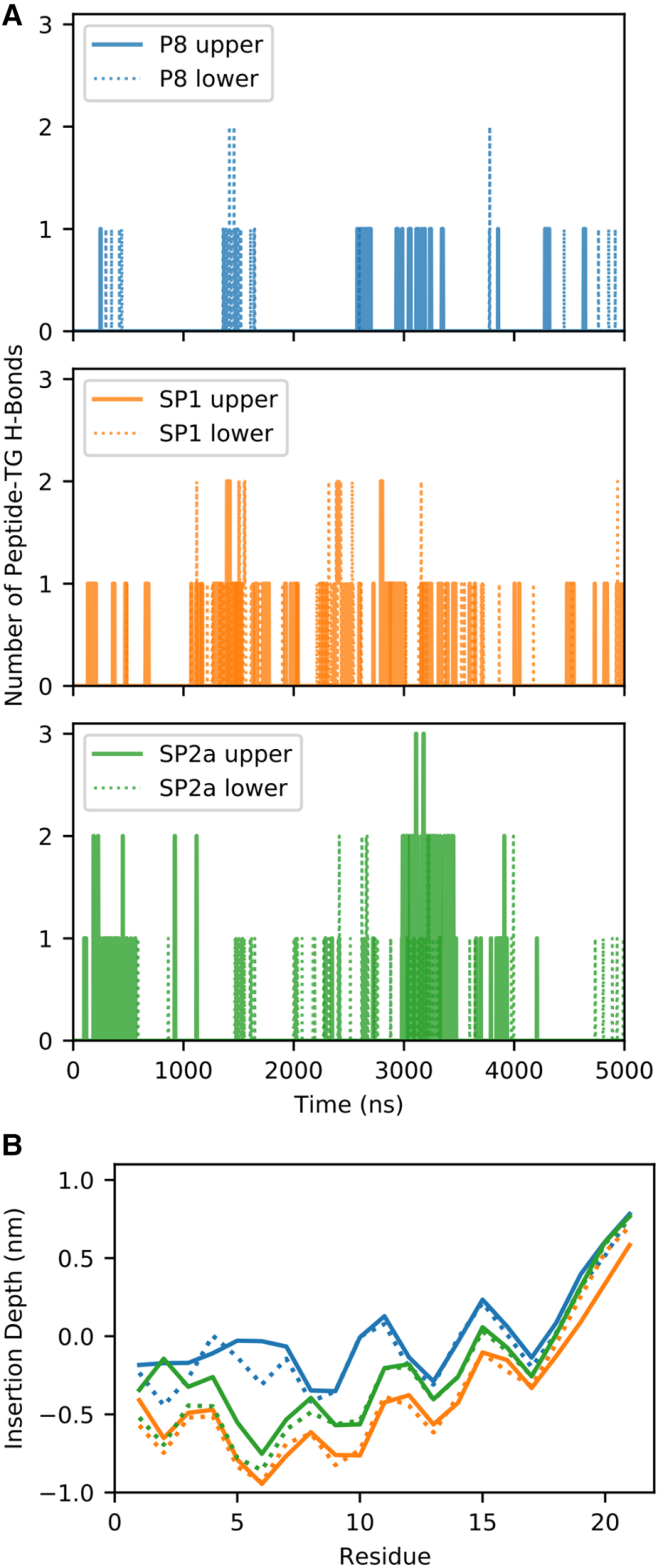
Quantification of molecular dynamic simulation of interaction of peptides with TG. (**A**) Number of hydrogen bonds (H-bonds) between the peptide and TG for the three different systems for the upper (solid line) and lower (dotted) monolayers. (**B**) Average insertion depth of the alpha carbon of each amino acid with respect to the phosphorus-plane (z = 0) of each system and monolayer.

During the simulation, TG molecules spontaneously diffuse between the core and the surface phospholipid monolayer and dynamically associate with monolayer-bound peptides. Figure 8 shows the 5 µs snapshots of the three simulation systems; see Supplementary Figures S4–S6 for additional time points. On average, P8 interacts with at least one TG molecule, whereas the modified stapled peptides directly interact with two or three TG molecules (Supplementary Figure S7). Direct interactions vary from transient association of hydrophobic regions to hydrogen bonding between the peptides and TG molecules. Timeseries analysis of the hydrogen bonding demonstrates a dynamic association behavior accompanied by a fluctuating insertion depth (Figure 9A). On average, P8 forms a hydrogen bond to TGs only 1.5% of the time, in contrast to 10.5% for SP1 and 8.7% for SP2a.

For a peptide to form a hydrogen bond to TGs in the core of the trilayer, it must be deeply inserted into the phospholipid monolayer to sufficiently prevent cradling by adjacent phospholipids, which typically block direct peptide-TG interactions. Indeed, compared to the native P8 peptide there is a much deeper insertion of the stapled or stitched peptides (Figure 9B). Moreover, for the modified peptides, the deepest amino acid insertion occurs for Thr6, a possible hydrogen bond donor, as opposed to P8 in which Ile8 and Gly9 residues showed the greatest depth of insertion. Notably, Ile8 (or Ile66 in the full-length ApoC2), which is thought to possibly directly interact with LPL, is less buried when compared Thr6 in the modified peptides. The greater relative degree of burying of Ile8 for P8 may partially account for its diminished ability to activate LPL. Furthermore, when considering the location of the critical amino acids for LPL activation identified in the Ala scan of D6PV (Y5, I8, D11 and Q12), all-atom simulations show that the hydrocarbon staple between position 3 and 10 restrains the conformation of the modified peptides to keep these key residues on the same polar face of the helix, which may facilitate their interact with LPL.

## Discussion

Our first generation of ApoC2 mimetic peptides utilized an artificial amphipathic helix called 18A (25, 75). 18A has no homology to ApoC2 or to any other exchangeable-type apolipoprotein (76) and would, therefore, likely be immunogenic, particularly after chronic use. Results from these early efforts, however, demonstrated the necessity for these peptides to bind to lipoproteins to stimulate lipolysis by LPL. In contrast to the first-generation peptides, D6PV is just based on the native ApoC2 sequence but does contain several amino acid substitutions in the random coil region of ApoC2, which may also make it immunogenic. Another major limitation of D6PV is its relatively long length (41 residues), which will make it difficult and expensive to manufacture in high yields. In addition, D6PV was not designed to be resistant to proteolysis, which may limit its half-life and make it unsuitable for oral delivery (77, 78). By using hydrocarbon staples and selective amino acid substitutions, we describe in this report a new generation of ApoC2 mimetic peptides that are much shorter and resistant to proteolysis.

Hydrocarbon stapling of peptides were first reported in 2,000 and now several therapeutic peptides with hydrocarbon staples are being tested in early-stage clinical trials (19, 79). They were first developed to stabilize alpha-helix formation, which are frequently involved in protein-protein interactions (80, 81). It was also discovered that hydrocarbon stapling of peptides facilitates their cellular internalization, and these peptides are now mostly being developed for blocking intracellular protein-protein interactions for cancer related targets (19, 82). We previously found that placement of hydrocarbon staples on the hydrophobic face of amphipathic peptides found on ApoA1 increase the ability of these peptides to bind lipids and to efflux cholesterol by the ABCA1 transporter (20). Based on the DMPC vesicle solubilization study, hydrocarbon staples on ApoC2 mimetic peptides likely act similarly in increasing the affinity of these peptides for lipids. The molecular dynamics simulations indicate that this may occur because of the interaction of the hydrocarbon staple with acyl chains in the phospholipid monolayer of lipoproteins. Furthermore, our findings from the Ala-scanning array suggested a possible new strategy for making the C-terminal helix of ApoC2 more amphipathic for facilitating its binding to lipids. Using this approach, we were successful in producing peptides about half the length of D6PV that contained either a single or double (stitched) hydrocarbon staple in the last helix of ApoC2.

Hydrocarbon staples are also known to protect peptides against proteolysis, because stabilization of helix formation decreases the ability of peptides to enter the active site of proteases (79). Interestingly, we observed the one Lys residue in the SP1 peptide, which is outside of the stapled region, was still resistant to trypsin. This may be due to extension of helix formation outside the stapled region because of hydrogen bonding across the peptide backbone with residues within and outside the stapled region. To further enhance the resistance of our peptides to proteolysis, we also investigated the use of several commonly used amino acid substitutions. Although not specifically tested in our study, the acetylation or the covalent attachment of longer chain fatty acids are known to block degradation by aminopeptidases (83, 84). Similarly, the modification of the C-terminus by amidation is known to block degradation by carboxypeptidases (85), but we did not use this modification in this study, because we previously found that the reduction of the net negative charge of these peptides reduces their solubility (16). Because our Ala-scanning studies indicated that the last 2 Glu residues are not critical for LPL activation, we instead replaced them with the D-stereoisomer of Glu, which is a known strategy for blocking proteolysis (86). The amino acid preceding the two terminal Glu residues is Gly, which likely increases the conformation flexibility of the C-terminus and makes its precise stereochemical configuration unimportant for LPL activation. Finally, we replaced the Gly19 with N-methyl-Gly (sarcosine) in the SP2 peptides. This was to further protect this peptide against trypsin cleavage, although this residue was already relatively resistant to trypsin in the SP1 peptide from the addition of the single hydrocarbon staple.

Both SP1 and SP2a appeared to as effectively lower TG and cholesterol in two different mouse models as the much longer D6PV peptide. A limitation of our study is that only the acute effect of a single dose of the peptides during the first 6 h on plasma lipids was monitored. Future studies to determine the half-life and other pharmacokinetic and pharmacodynamic properties of the modified stapled peptides are required to determine if hydrocarbon stapling provides additional advantages over D6PV. Multiple dose type studies will also be needed to determine if the effectiveness of the peptides may change with repeated use.Additional formulation work will also be needed to try to develop our peptides as oral agents, although recent advances in using cell permeation enhancers like Sodium Salcaprozate (87) and Labresol® (88) to make other peptides that are much longer orally available will facilitate this work. Our results from the Ala-scanning array and molecular dynamic simulation also suggest a future strategy for making additional substitutions in the polar face of the last helix of ApoC2 for further improving LPL activation. Interestingly, some of the amino acids on the polar face of the last helix are fairly hydrophobic (e.g., Ile8). It is possible that these residues help form a hydrophobic channel to deliver TG transferred from the hydrophobic core of TRL to the active site of LPL. If so, the observations from our molecular dynamic simulations that the SP1 and SP2a peptides penetrate deeper into lipoproteins and directly interact with TG, while preventing Ile8 from pointing toward the hydrophobic core, could explain their improved activity. The binding of stapled ApoC2 mimetic peptides may also enhance delivery of TG to the active site by a rotation type mechanism, possibly in conjunction with the hydrophobic residue Ile8, like has been proposed for the C-terminus of ApoC2 protein (89), or simply by disturbing the lipid packing of the phospholipid monolayer. Additionally, ApoC2 has recently been shown to bind to a similar region of LPL as ANGPTL4 (90–92), thus apoC2 mimetic peptides could also possibly stabilize LPL against inactivation by ANGPTL4. Another interesting possibility is that ApoC2 mimetic peptides may also counter the anti-lipolytic effects of ANGPLT3/8, an already established therapeutic target that have shown great effect in lowering LDL-C and ApoB even in Familial Hypercholesterolemia patients (93, 94). Further progress in identifying the mechanism of action of ApoC2 and how it interacts with LPL should provide a rationale for the future design of ApoC2 mimetic peptides.

In summary, we describe a new generation of ApoC2 mimetic peptides that contain hydrocarbon staples, which allows for the synthesis of much shorter peptides that are not only more potent but also more resistant to proteolysis. Additional optimization of these peptides may ultimately lead to a new therapy for genetic ApoC2 deficiency, and for decreasing residual ASCVD risk after statin treatment in hypertriglyceridemic patients.

## Data Availability

The raw data supporting the conclusions of this article will be made available by the authors, without undue reservation.
