# Supplementary figures and images for: Short hydrocarbon stapled ApoC2-mimetic peptides activate lipoprotein lipase and lower plasma triglycerides in mice
Source: Front Cardiovasc Med. 2023 Jul 21;10:1223920. doi: 10.3389/fcvm.2023.1223920 (PMC10403075; doi:10.3389/fcvm.2023.1223920)

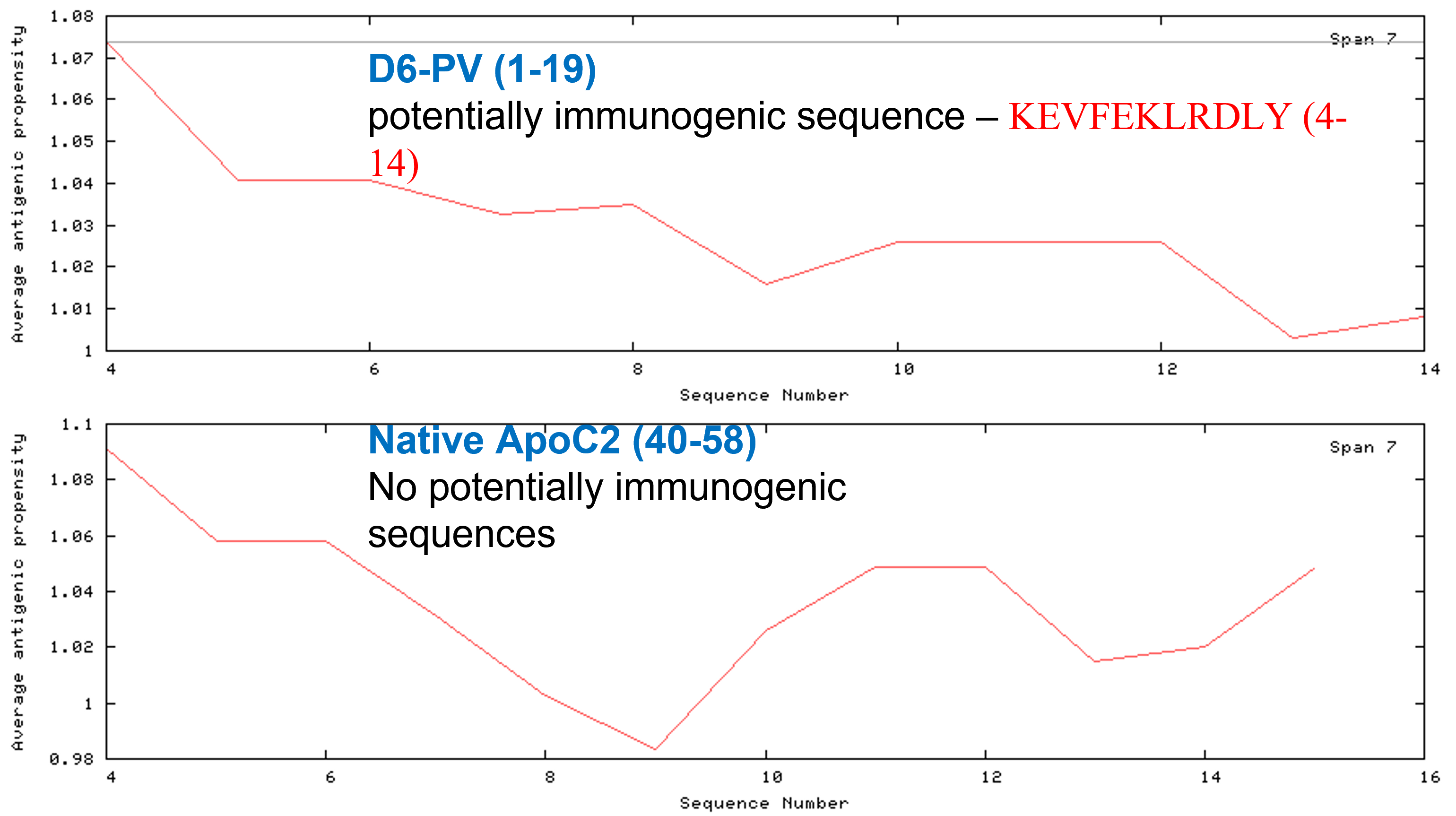

Supplement: Supplementary file 1 [file Datasheet1.zip › Suppl. fig 1 .tif]

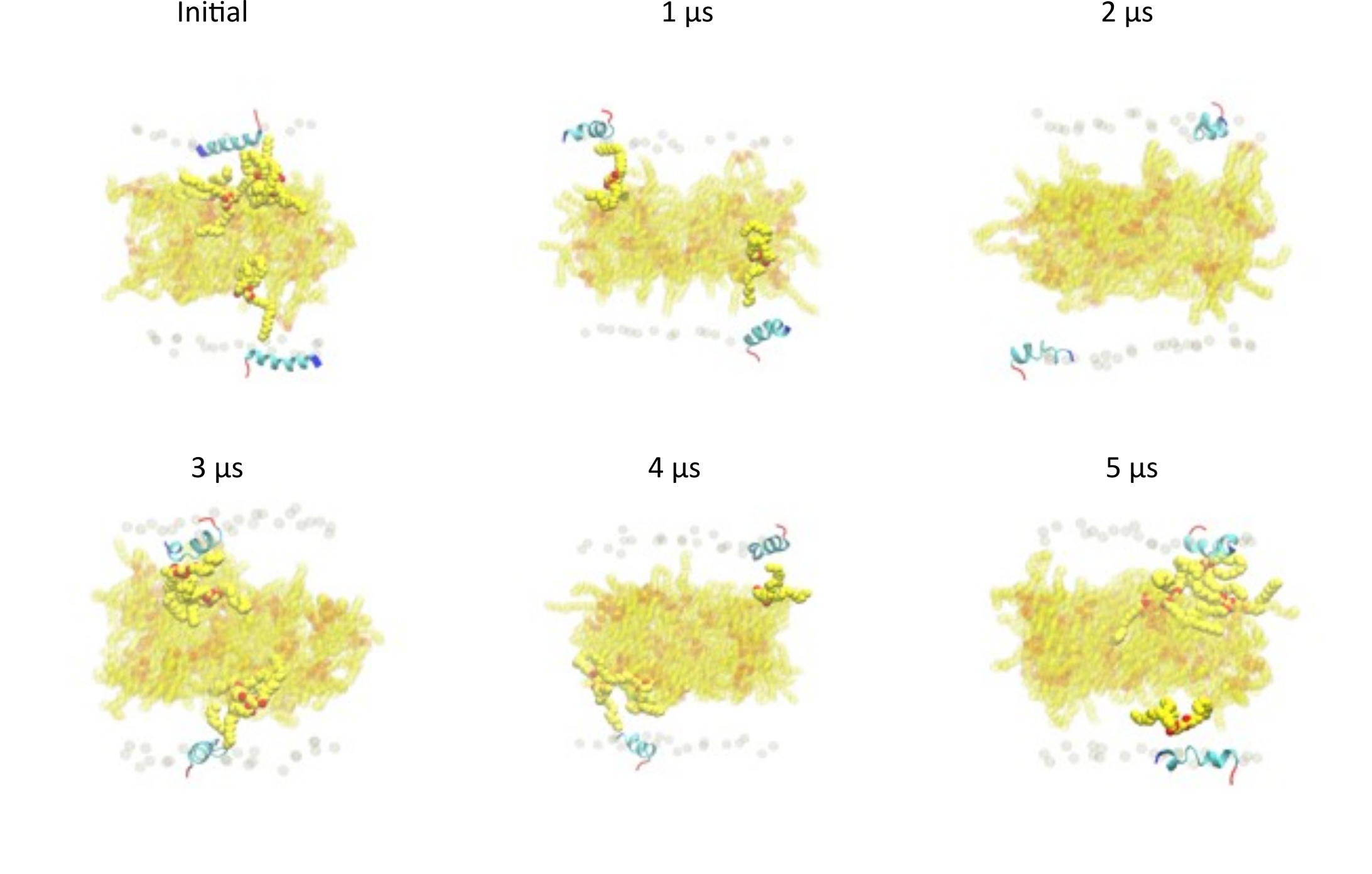

Supplement: Supplementary file 1 [file Datasheet1.zip › suppl. fig,4.tiff]

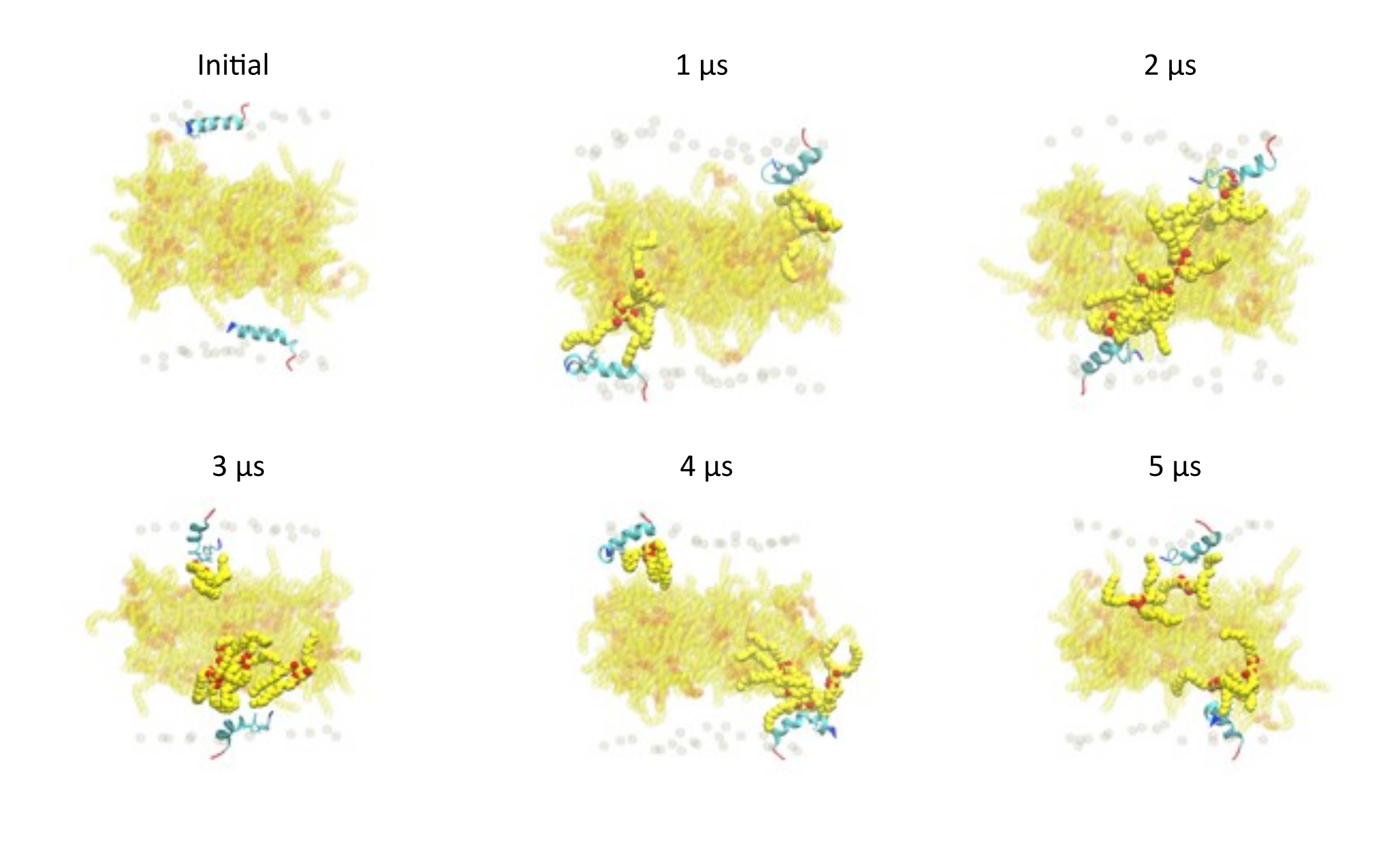

Supplement: Supplementary file 1 [file Datasheet1.zip › suppl. fig,5.tiff]

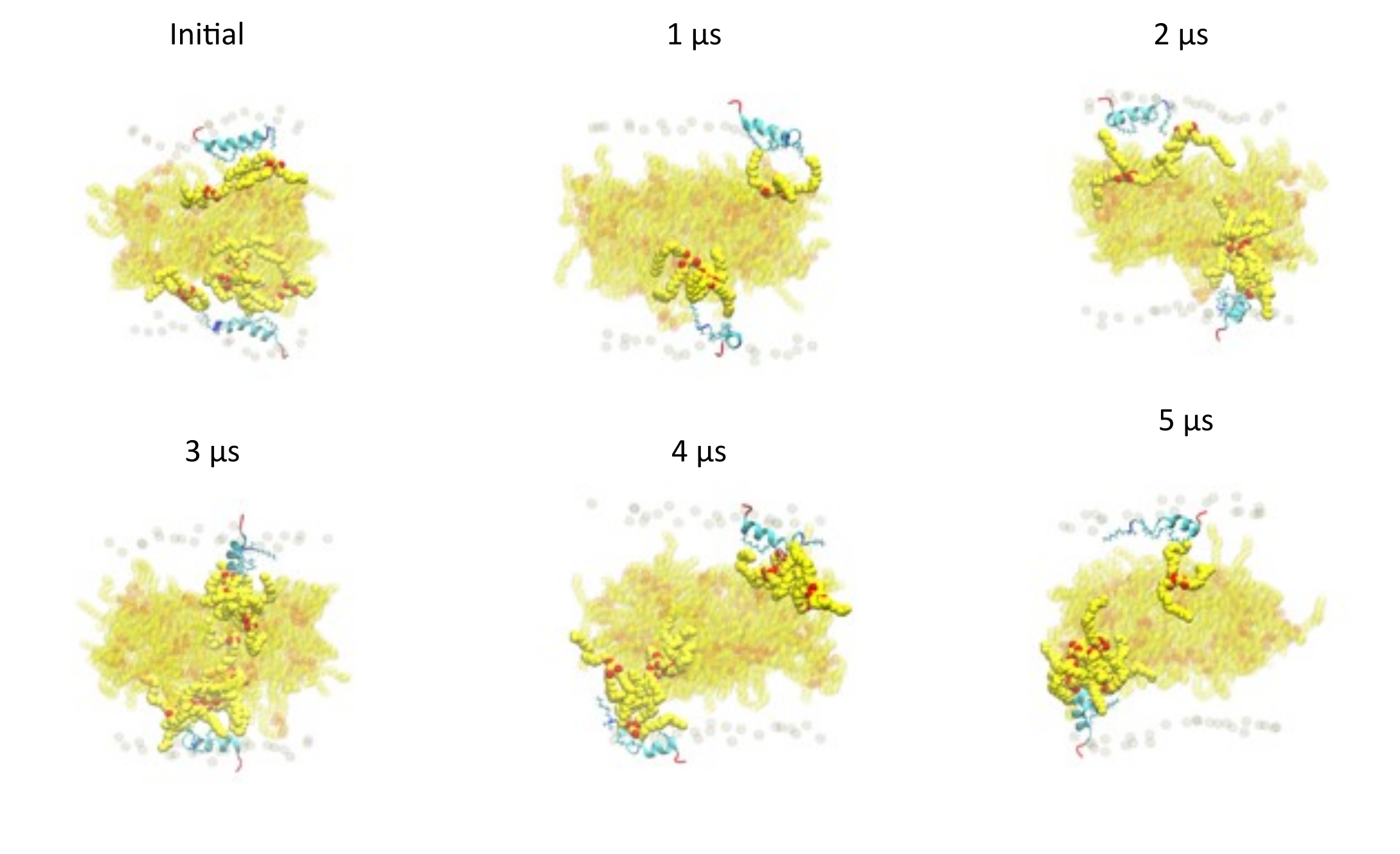

Supplement: Supplementary file 1 [file Datasheet1.zip › suppl. fig,6.tiff]

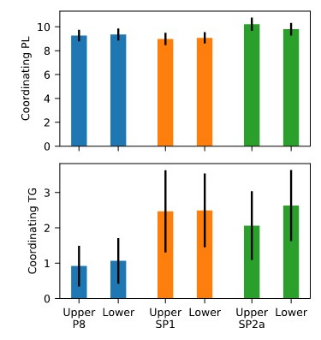

Supplement: Supplementary file 1 [file Datasheet1.zip › suppl. fig.7.tiff]

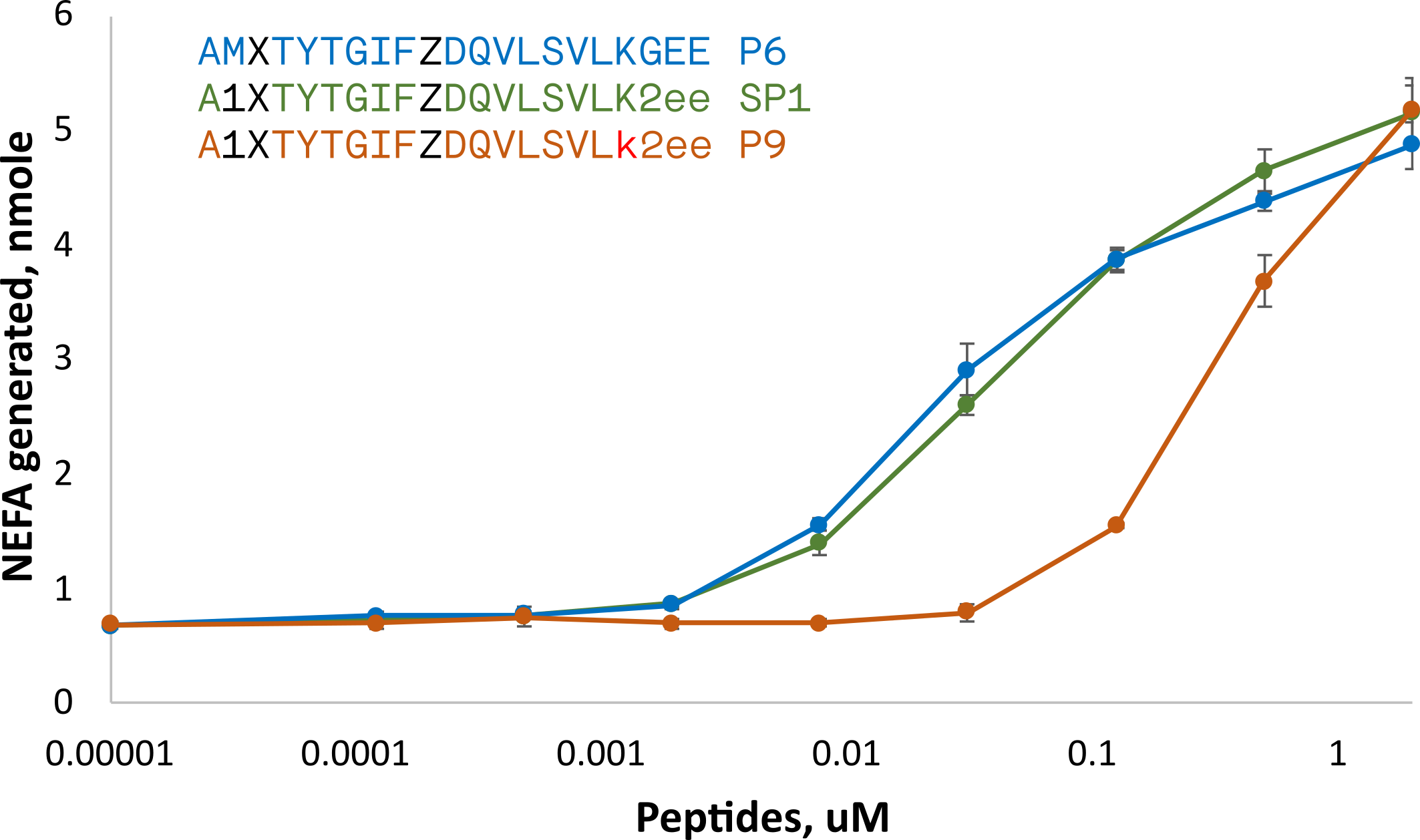

Supplement: Supplementary file 1 [file Datasheet1.zip › Suppl.Fig.2.tiff]

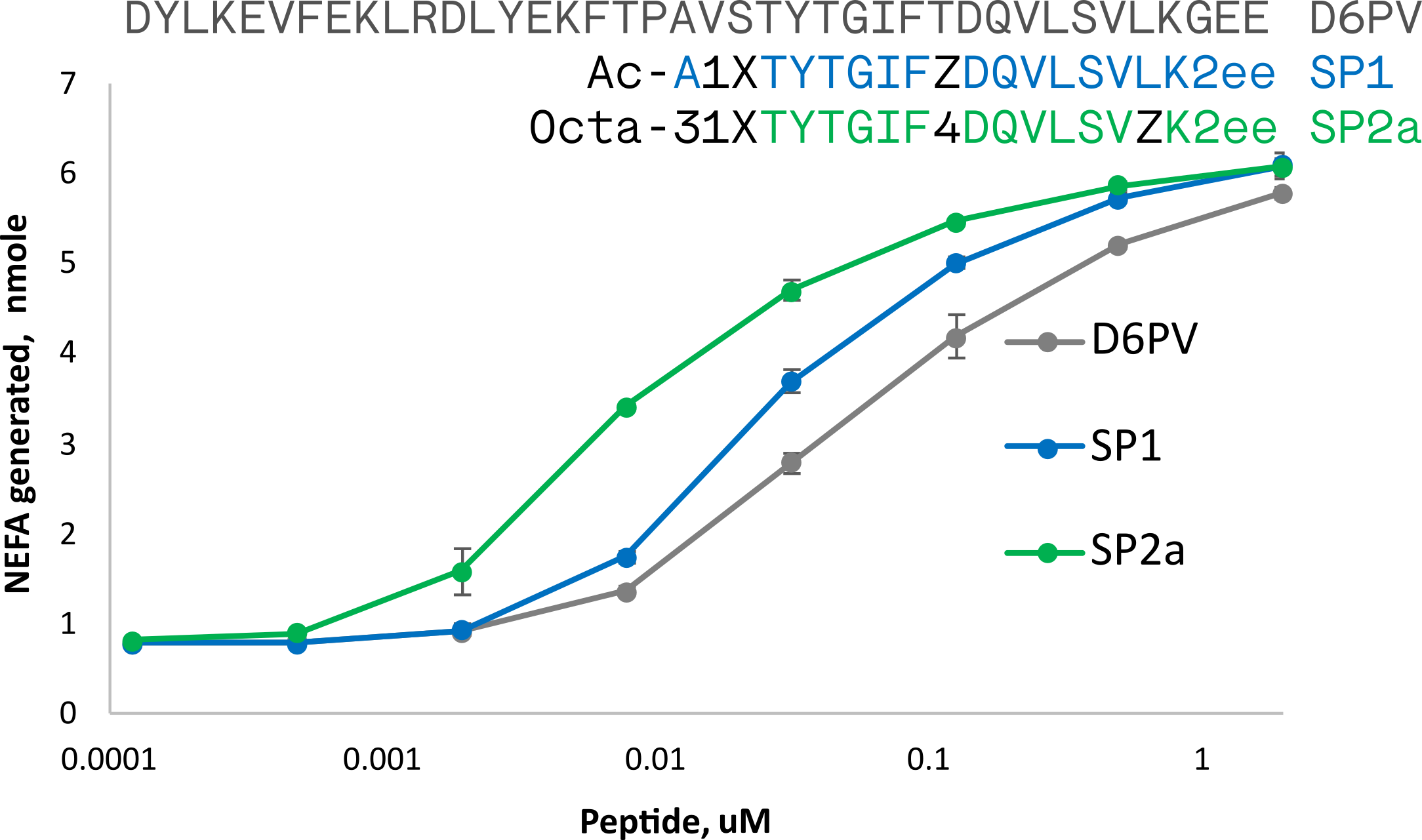

Supplement: Supplementary file 1 [file Datasheet1.zip › Suppl.Fig.3.tiff]
