# Supplementary material for: Short hydrocarbon stapled ApoC2-mimetic peptides activate lipoprotein lipase and lower plasma triglycerides in mice
Source: Front Cardiovasc Med. 2023 Jul 21;10:1223920. doi: 10.3389/fcvm.2023.1223920 (PMC10403075; doi:10.3389/fcvm.2023.1223920)
Supplement: Supplementary file 2 [file Table1.docx]

Short Hydrocarbon Stapled ApoC2-mimetic Peptides Activate Lipoprotein Lipase and Lower Plasma Triglycerides in Mice

**Supplementary Figure 1. Antigenic plot for the first helix of D6PV and matching sequence of native ApoC2.** The antigenic propensity of the sequence is plotted over the amino acid sequence number. Antigenic determinant sequence of D6-PV is indicated on the graph.

**Supplemental Figure 2. Peptide dose response curves for activation of LPL.** For P9 peptide Lys18 was replaced with its D-isomer (small k) and compared to P6 and SP1 for their ability to activate LPL as described in Fig. 2. The color code of the peptide sequences (see legends) are matched to the color of the dose response curves. Amino acid identify are indicated by single letter abbreviation. X indicates position of (R)-α-methyl,α-octenyl-glycine and Z indicates position of (S)-α-methyl,α-pentenyl-glycine and are the two amino acid substitutions made to introduce the two different arms of the hydrocarbon staples. 1= Norleucine; 2= N-methyl-glycine. Results represent the mean ± 1SD of triplicates.

**Supplemental Figure 3. Peptide dose response curves for activation of LPL.** Indicated substitutions were made for SP2a and its in vitro ability activate LPL was compared to SP1 and D6-PV. The color code of the peptide sequences (see legends) are matched to the color of the dose response curves. Amino acid identify are indicated by single letter abbreviation. 1= Norleucine; 2= N-methyl-glycine, 3= amino-isobutyric acid, 4= bis-pentenyl-glycine (B5), X= (R)-α-methyl,α-octenyl-glycine; Z= (S)-α-methyl,α-pentenyl-glycine; Staples are between X, 4 and Z positions. Results represent the mean ± 1SD of triplicates.

**Supplemental Figure 4. Molecular dynamic simulation of interaction of P8 peptide with TG.** Timeseries of P8 system. Coloring: Peptide N- and C-termini blue and red, respectively, with remaining residues cyan; carbons and hydrogens of peptide staple and acylation cyan and white, respectively; nearby phosphorus atoms of PL gold spheres; carbon and oxygen of TG in yellow and red spheres, respectively. TG with any atom near peptide is opaque and background TG are semi-transparent.

**Supplemental Figure 5.** **Molecular dynamic simulation of interaction of SP1 peptide with TG.** Timeseries of SP1 peptide system. Coloring: Peptide N- and C-termini blue and red, respectively, with remaining residues cyan; carbons and hydrogens of peptide staple and acylation cyan and white, respectively; nearby phosphorus atoms of PL gold spheres; carbon and oxygen of TG in yellow and red spheres, respectively. TG with any atom near peptide is opaque and background TG are semi-transparent.

**Supplemental Figure 6.** **Molecular dynamic simulation of interaction of SP2a peptide with TG.** Timeseries of SP2a peptide system. Coloring: Peptide N- and C-termini blue and red, respectively, with remaining residues cyan; carbons and hydrogens of peptide staple and acylation cyan and white, respectively; nearby phosphorus atoms of PL gold spheres; carbon and oxygen of TG in yellow and red spheres, respectively. TG with any atom near peptide is opaque and background TG are semi-transparent.

**Supplemental Figure 7. Quantification of the interaction of peptides with lipids.** Number of phospholipids (PL) (upper panel) and TG (lower panel) molecules coordinating with indicated peptide in the upper and lower compartments of the model trilayer.
